# Left atrial appendage closure device embolization—You may not know if you do not look

**DOI:** 10.1016/j.hrcr.2023.07.001

**Published:** 2023-07-13

**Authors:** Dan Musat, Suneet Mittal

**Affiliations:** Valley Hospital, Ridgewood, New Jersey

Advancements in device technology led to marked therapeutic advances in interventional cardiology and electrophysiology. Adequate fixation to moving cardiac structures have been an Achilles’ heel of intracardiac implantable devices; the consequence is a relatively small but clinically highly significant risk of device migration or frank embolization, with potential catastrophic consequences.

Percutaneous left atrial appendage occlusion (LAAO) is an alternative for stroke prevention for patients with atrial fibrillation introduced more than 20 years ago, and has since seen an exponential growth, with more than 300,000 devices implanted worldwide. Currently, in the United States, the 2 FDA-approved devices—namely, the Watchman FLX (Boston Scientific, Marlborough, MA) and Amplatzer Amulet (Abbott, Chicago, IL)—are recommended for patients who are at high risk of thromboembolism but are poor candidates for long-term anticoagulation.

With first-generation LAAO devices, a device embolization (DE) rate of 0–2% was reported.^1^^,^^2^ Lessons learned from the first-generation Watchman 2.5 led to enhancements with the second-generation device (Watchman FLX). The design changes were geared to increase the safety of implant, increase device stability, and minimize the risk of embolization. The positive impact of these designed changes was validated in the Pinnacle FLX study that led to FDA approval of the device in the United States, in which no DE were reported.^3^

In this issue of the journal, Bhagat and colleagues^4^ report a case of a 73-year-old man with asymptomatic embolization of a 27 mm Watchman FLX device into the left ventricular outflow tract, detected on scheduled transesophageal echocardiography (TEE performed 35 days after implant. The description of the implant procedure is limited by the fact that the implant procedure occurred at a different center than where the embolization was recognized. The authors provide preimplant TEE images, which reveal a chicken-wing morphology; of note, no preprocedural computed tomography scan was performed. The implant procedure was reported as being uneventful, with only 1 deployment that met adequate PASS criteria (position, anchoring, size, and seal). The device size was adequate for the reported dimensions. However, since the authors were not part of the implanting facility, several significant procedural details are not available, such as the rhythm at implant, the left atrial pressure, postimplant images, and degree of compression observed—all important to understand the possible causes of DE in this patient.

Interestingly enough, the patient reported no postprocedure symptoms, and a previously implanted loop recorder showed no arrhythmias. The patient underwent successful surgical retrieval of the device and left atrial appendage ligation after being transferred back to the implanting facility. No further details of the findings are available.

This case report might initially engender surprise given the Pinnacle FLX data, and the authors describe this as the first case reported of Watchman FLX embolization in the literature. However, a deeper dive into the literature disclosed at least 2 other case reports from European centers of Watchman FLX embolization. This included an asymptomatic late embolization, discovered at 3 months postprocedure TEE to be in the left atrium; the device was successfully retrieved percutaneously.^5^ In the other case, embolization occurred immediately postprocedure to the abdominal aorta and was associated with limb ischemia; this device was also retrieved percutaneously. However, the extraction was complicated by thrombotic occlusion of popliteal artery and aortic rupture with hemorrhagic shock, both successfully treated.^6^

There are also several analyses of real-world experience from the NCDR LAAO Registry^7^, ^8^, ^9^ reporting a very low rate of Watchman FLX embolization. The SURPASS analysis of 16,048 patients,^8^ presented at the Cardiovascular Research Technologies meeting in 2022, reported an incidence of DE of 0.01% (2 patients) at discharge and 0.03% (5 patients) at 45 days. More recently, 2 large series, by Friedman and colleagues^7^ and Price and colleagues,^9^ reported a 50%–65% lower incidence of predischarge device migration or embolization (DME) with Watchman FLX compared to first-generation device: 0.04% vs 0.08% (*P* = .0048) and no events after discharge in the first series and 0.02% vs 0.06% (*P* = .028) in the second one. However, the greatest number of Watchman FLX DE, 42 cases, is reported by Velagapudi and colleagues^10^ in the analysis from the FDA MAUDE database between August 2021 and February, 2022. A personal search of the MAUDE database from March 2022 until May 2023^11^ yielded another 36 reports of DE, mostly occurring prior to discharge, likely related to procedural technique, and usually symptomatic (arrhythmia, ischemia, or hemodynamic instability). The location with highest incidence of embolization was the aorta (38%), followed by the left atrium (18%) and the left ventricle (7%).^10^ The current case presents a rare scenario of late DE (although no imaging was performed prior to discharge) in the ventricle, asymptomatic and with no recorded arrhythmia on continuous monitoring.

Although the cause of DE is not always clear, there are several anatomic (incorrect sizing, low left atrial pressure, wide left atrial appendage [LAA] neck, and high LAA velocity), procedural (off axis, failure to engage the hooks, cardioversion, cough), and device (inappropriate sheath coaxiality, cable malfunction) factors reported to predispose it.^1^ Best insights of predictors for DE were reported on a cohort of 120,278 Watchman procedures from the NCDR LAAO Registry,^7^ with 84 DME occurring in hospital and 54 DME after discharge. The in-hospital DME was higher at hospitals with lower procedural volume, with first-generation Watchman, larger LAA ostia, and a smaller difference between device and LAA ostial size. Postdischarge, men, taller and heavier patients, and patients with sinus rhythm at implant had higher risk of DME. Importantly, the report showed a high mortality of 14% with in-hospital DME (20.5% when surgery required), which decreased to 3.7% in postdischarge DME. Risk features of the case reported in this issue of the journal^4^ included male sex, presumed sinus rhythm at implant, and a more complex chicken-wing anatomy; however, other pertinent information might be missing.

Once a DE is identified, the extraction modality depends mostly on where the device is embolized and hemodynamic stability. For devices in the ventricle and those causing hemodynamic instability, surgical retrieval is preferred.^2^ The devices in the left atrium and aorta are often successfully retrieved percutaneously. In the described case, the patient appropriately underwent successful surgical device retrieval.

The accumulated experience led to SCAI/HRS Expert Consensus recommendations on LAAO procedures including physician and institutional requirements, preprocedural and intraprocedural imaging, safe and effective technical aspects, and predischarge imaging with echocardiogram to rule out pericardial effusion and DE.^2^ It is less clear what the timing and modality of outpatient imaging should be for possible detection of DME, especially now with immediate postimplant dual antiplatelet strategy and the trend for postponing the first postimplant imaging to 3 months, as seen in the latest ongoing OPTION and CHAMPION trials.

In summary, the Watchman FLX is not immune to embolization; however, the incidence is very low and is much improved compared with Watchman 2.5. Most of the cases occur prior to hospital discharge and are due to improper implant technique. Best practice is prevention with preprocedural planning and careful implant technique. However, we are not yet ready to abandon postprocedure imaging because you may not know if you do not look.
